# “One Note Higher”: A Unique Pediatric Hand Fracture

**DOI:** 10.5811/cpcem.2021.3.51806

**Published:** 2021-05-06

**Authors:** Scott Szymanski, Michael Zylstra, Aicha Hull

**Affiliations:** Madigan Army Medical Center, Department of Emergency Medicine, Tacoma, Washington

**Keywords:** Extra-octave, Salter-Harris type II, fracture, pencil method, 90-90 method

## Abstract

**Case Presentation:**

An otherwise healthy, 12-year-old male presented to the emergency department after a fall down the stairs in which he landed on his right hand. Radiographs demonstrated a Salter-Harris II fracture at the base of the proximal phalanx of the fifth digit with ulnar deviation, also known as an “extra-octave“ fracture. Orthopedic surgery was consulted and the fracture was reduced and placed in a short-arm cast. The patient was discharged and scheduled for orthopedic follow-up.

**Discussion:**

A Salter-Harris II fracture at the base of the proximal phalanx of the fifth digit with ulnar deviation is referred to as an “extra-octave” fracture due to the advantage a pianist would gain in reach of their fifth phalanx if not reduced. However, reduction is needed if the fracture is displaced and can be achieved by several described methods including the “90-90” or “pencil” methods followed by cast or splint application. Percutaneous pinning is rarely needed. Complications include flexor tendon entrapment, collateral ligament disruption, and malunion leading to a “pseudo-claw” deformity. We recommend that all extra-octave fractures receive orthopedic follow-up in one to two weeks or sooner if severely displaced.

## CASE PRESENTATION

**Image 1:**
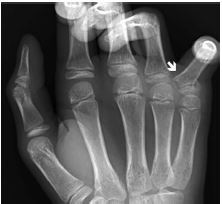
Radiograph with white arrow pointing to ulnar deviation of the fifth metacarpophalangeal due to an “extra-octave” fracture.

A 12-year-old male presented to the emergency department (ED) after falling awkwardly on his right hand. The patient experienced immediate pain and an obvious deformity of the fifth digit. He had increased pain with active and passive movements but denied altered sensation. Exam showed swelling, ulnar displacement, and decreased range of motion at the fifth metacarpophalangeal (MCP) joint. Capillary refill and sensation were normal throughout. Radiographs showed an “extra-octave” fracture (Image 1).

An “extra-octave fracture” is a Salter-Harris II fracture at the base of the fifth proximal phalanx with ulnar deviation. A digital block was performed followed by reduction using traction and adduction, using the provider’s finger as a fulcrum. The patient was placed in a short-arm cast and scheduled for orthopedic follow-up.

## DISCUSSION

**Image 2:**
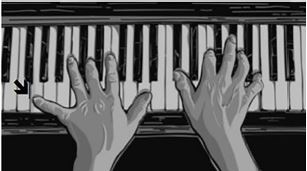
A pianist striking an “extra-octave” (arrow), possibly due to inadequate treatment of a remote fracture.^4^ (Open access article reprinted with written permission from the authors).

**Image 3:**
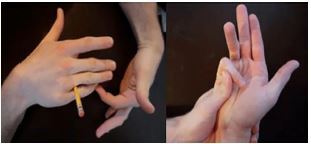
The “pencil” (left) and “90-90” (right) methods of reduction.^4^ (Open access article reprinted with written permission from the authors).

Hand fractures make up more than 2% of all ED visits in the pediatric population.^1^ Given this high incidence, emergency physicians will treat many of these fractures. Thus, proper identification and management of higher risk injuries is necessary. An “extra-octave” fracture is a type of Salter-Harris II fracture at the base of the fifth proximal phalanx with ulnar deviation, although some classify it as a juxtaepiphyseal II fracture rather than Salter-Harris II.^2^,^3^ The term “extra-octave” fracture refers to the advantage a pianist would gain in reaching an “extra octave” if his fracture was not reduced (Image 2).^4^ It is the most common fracture type at the proximal phalanx in children, occurring at a mean age of 10 years.^2^–^4^ Reduction can be best achieved by using the so-called “pencil” or “90-90” methods (Image 3), with similar outcomes.^4^

The former is accomplished by placing a pencil in the web space of the fourth and fifth digits and using it is a fulcrum while applying traction, mild flexion, and adduction at the MCP joint (Image 3, left). The “90-90” method involves flexing the MCP and applying force in a volar direction on the metacarpal shaft and a dorsal direction on the proximal interphalangeal (PIP) joint (Image 3, right). Care must be taken during reduction and immobilization to ensure the finger is not trapped in residual extension. This can lead to a “pseudo-claw” deformity, in which with active extension, the finger is deformed in 10–15 degrees of hyperextension at the MCP and 10–15 degrees of flexion at the PIP joint. To avoid this, Al-Qattan recommends immobilization with an ulnar gutter splint or cast and the MCP flexed at 90 degrees.^5^ Other complications of the more severely displaced “Type II” fractures include flexor tendon entrapment and collateral ligament disruption.^5^ These fractures can make closed reduction difficult and necessitate more advanced orthopedic intervention such as open reduction.^5^ Overall, most patients do not require surgery and will regain full range of motion.^4^

**CPC-EM Capsule**

What do we already know about this clinical entity?

*An extra octave is a type of Salter Harris II fracture named for the advantage a pianist would gain in reaching an “extra octave” if not adequately reduced.*

What is the major impact of the image(s)?

*The ulnar displacement associated with this fracture may require special reduction techniques to achieve optimal re-alignment.*

How might this improve emergency medicine practice?

*Knowledge of this specific fracture, these reduction techniques, and the appropriate management of this injury may help minimize associated complications.*

To date, no report of this type of fracture has appeared in the emergency medicine literature. Based on the review of the available orthopedic and pediatric literature, we recommend that all “extra-octave” fractures receive orthopedic follow-up in one to two weeks, and the more severe “Type II” fractures receive more expeditious follow-up to avoid the complications described above.
